# Middle Fossa Dura Implantation of the Bonebridge in Children With Craniofacial Malformations: A Case Report

**DOI:** 10.7759/cureus.107960

**Published:** 2026-04-29

**Authors:** Ana Sousa Menezes, Miguel Breda, Filipa Ferreira, Mariana Gouveia, Daniela Ribeiro, Luís Dias

**Affiliations:** 1 Department of Otorhinolaryngology - Head and Neck Surgery, Hospital de Braga, Braga, PRT

**Keywords:** active transcutaneous bone conduction device, auditory implant, bone conduction hearing device, hearing rehabilitation, med-el bonebridge, middle fossa dura

## Abstract

Hearing rehabilitation in children with craniofacial malformations is challenging not only because of reduced bone thickness but also due to complex anatomical and aesthetic considerations related to reconstruction. In such cases, conventional mastoid or retrosigmoid implantation of active transcutaneous bone conduction devices may not be feasible.

We present our experience with two clinical cases, in which the optimal surgical site for placing a transcutaneous bone conduction device was over the middle fossa dura, selected after careful radiological assessment of skull thickness and venous anatomy.

This technique proved to be a safe and effective alternative in children with limited bone thickness and/or craniofacial malformations, offering satisfactory hearing results and favorable cosmetic outcomes. The middle fossa dura placement may represent a valuable option for expanding indications of active transcutaneous bone conduction implants (t-BCIs) in complex pediatric cases.

## Introduction

The functional hearing rehabilitation in children with congenital external auditory canal atresia has always been a challenge for many reasons. Congenital external auditory canal atresia, or congenital aural atresia, is defined by the partial or complete lack of development of the external auditory canal [1]. It affects 1 in every 10,000 to 20,000 births, with unilateral cases being seven times more common than bilateral cases [1]. It may occur in isolation or be associated with microtia and middle ear anomalies, and may also be part of syndromes such as Treacher-Collins, Goldenhar, or Crouzon [1]. Due to its distinct embryological origin, inner ear malformations are less frequently associated [1]. The treatment of children with congenital aural atresia involves both functional management of the hearing loss (HL) - typically conductive - and aesthetic correction of the pinna malformation, when present.

For the latter, reconstructive surgery using autologous rib cartilage is an option, usually postponed until after the age of 10 [2]. Alternatively, earlier reconstruction can be performed using pre-formed synthetic polyethylene frameworks [2]. Implant-retained auricular epitheses also offer an aesthetic reconstruction option and are used routinely [3].

Functional rehabilitation of hearing in congenital aural atresia is crucial to prevent adverse effects on language development, verbal communication, social-emotional skills, and academic performance. Although often left untreated in the past, the detrimental impact of unilateral HL on speech perception in noise and sound localization is now well recognized [4]. Therefore, it is imperative to address both unilateral and bilateral HL in children with congenital aural atresia. This rehabilitation can include different options. Traditional reconstructive surgery of the ear canal and middle ear typically combines canaloplasty and tympanoplasty. However, this approach has become less favored due to the intraoperative risk of injuring an aberrant facial nerve, the unpredictable functional outcomes of ossicular reconstruction, and the potential for postoperative re-stenosis of the ear canal [5,6].

The use of bone conduction hearing aids integrated into elastic softbands, fixed on a headband or eyeglasses, is initially indicated to treat conductive HL in young children. Although these devices may not be well tolerated due to discomfort from head pressure, sweating, or aesthetic stigma, more recently, the introduction of implantable hearing devices has progressively influenced both the indications and timing of surgical intervention. Bone conduction implantable hearing devices are classified as percutaneous or transcutaneous.

Percutaneous bone conduction implant (p-BCI) consists of a processor that converts sound into vibrations, which are transmitted to a pillar abutment across the skin surface that is screwed and osseointegrated into the skull bone [5]. Although major complications are rare, skin reactions and infections at the implant site have been reported in 2.4% to 38.1% of cases. Failure of osseointegration and implant extrusion have been documented in up to 18% of adults and 14.3% of children, with an overall revision rate ranging from 1.6% to 17.4% in adults and 0% to 25% in children [5,7].

Transcutaneous bone conduction implants (t-BCI) were developed to overcome these limitations. In these, the external processor connects to the subcutaneous implant via magnetic coupling, leaving the skin intact [5].

The Bonebridge is an active t-BCI system (MED-EL, Innsbruck, Austria), which requires subcutaneous placement of a receiver and stimulator, connected to a vibrating bone conduction floating mass transducer (BC-FMT). The BC-FMT is drilled into the mastoid bone and secured with two screws [5]. However, in some children with craniofacial malformations, conventional implantation sites are not feasible. In these cases, alternative approaches, such as middle fossa placement, may be considered.

We report two pediatric cases of functional rehabilitation using the Bonebridge for unilateral external auditory canal atresia - one in a patient with a normal pinna and another in a patient with microtia.

## Case presentation

Case 1

Patient Information

A seven-year-old female was referred to our department for hearing rehabilitation. She had a history of Goldenhar syndrome with craniofacial involvement and had previously undergone right mandibular reconstruction using a costochondral graft at the age of four. Her main complaint was bilateral HL, with reported difficulties in daily communication.

Clinical Findings

**Figure 1:**
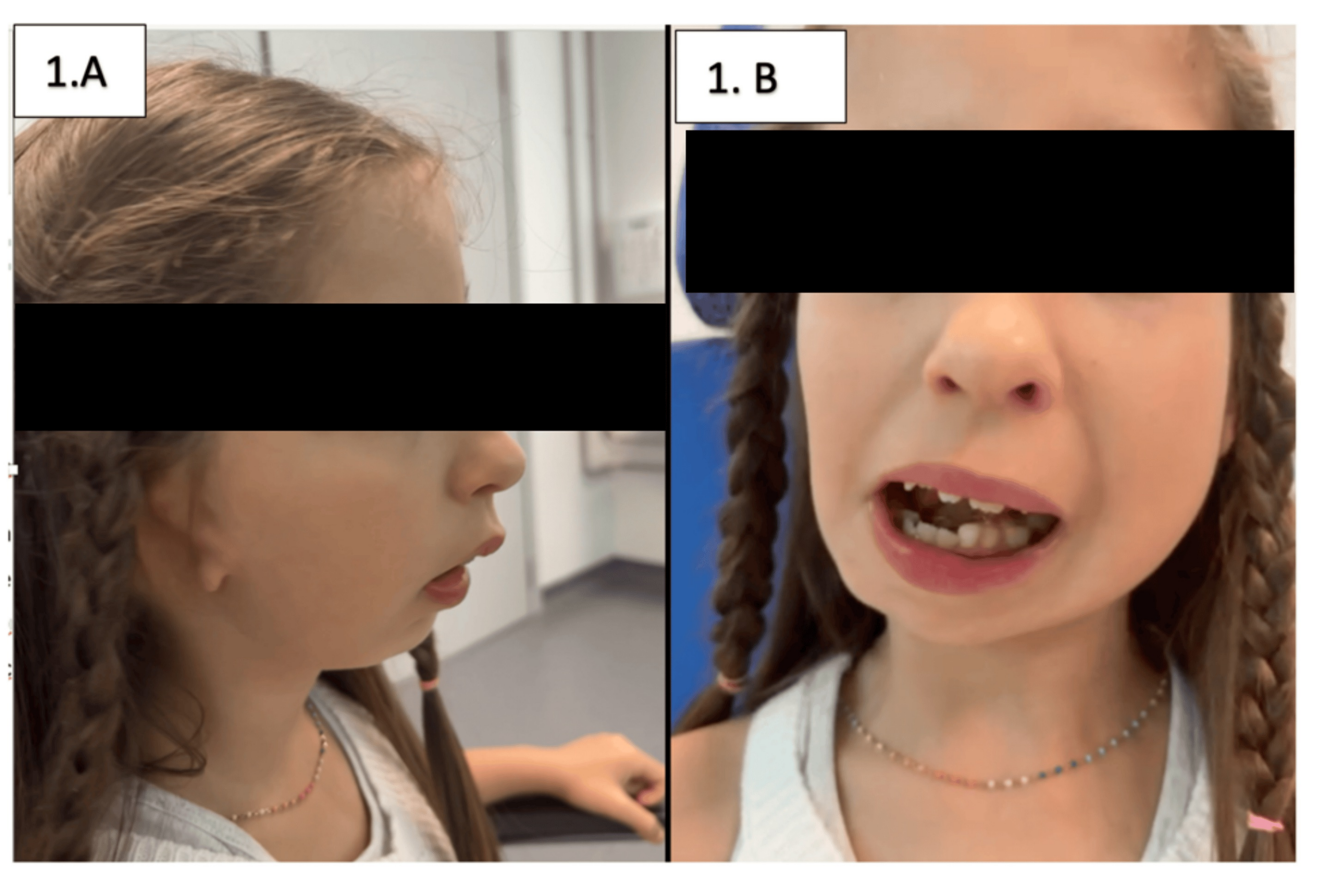
(A and B) Patient 1, a seven-year-old female with Goldenhar syndrome. She presented with right-sided hemifacial microsomia, ipsilateral microtia, and malocclusion due to mandibular deviation. The patient provided written and signed consent, allowing publication of this identifiable facial image in an open-access journal.

Physical examination revealed right-sided hemifacial microsomia, ipsilateral microtia, and mandibular deviation with malocclusion (Figure 1). Otoscopy of the left ear showed signs of effusive otitis media.

Diagnostic Assessment

Audiometric testing demonstrated bilateral conductive HL, severe on the left side and mild on the right.

The pure tone average (PTA), measured at 0.5, 1.0, 2.0, and 4.0 kHz, was 62.5 dB HL in the left ear and 20.5 dB HL in the right ear. Speech recognition thresholds were 65 dB HL in the left ear and 20 dB HL in the right ear, with 100% maximum discrimination bilaterally.

**Figure 2:**
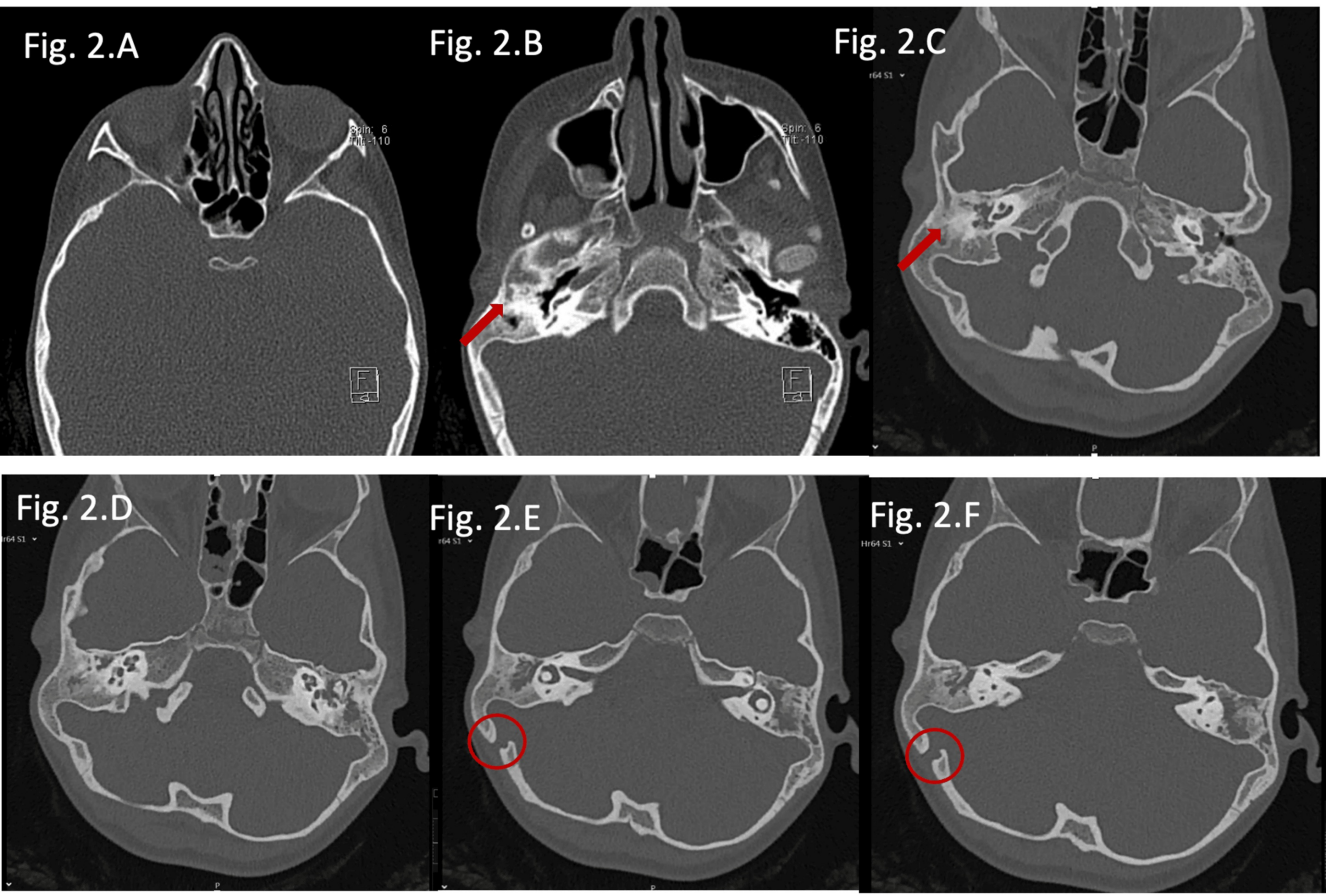
(A-F) Temporal bone CT scan (axial section) of Patient 1. An external auditory canal atresia (red arrow) and microtia can be seen, as well as a middle ear malformation. A very thin skull bone (2A) and a large right emissary vein posteriorly (red circle) are also evident.

Temporal bone computed tomography confirmed external auditory canal atresia and microtia (Figures 2B-2C). Additionally, it revealed markedly reduced temporal bone thickness in the middle fossa region (Figure 2A) and the presence of a prominent emissary vein (Figures 2E-2F), limiting safe implantation in conventional sites.

Surgical Proposal

Given the unfavorable mastoid anatomy, reduced bone thickness, and the need to preserve tissue for future auricular reconstruction, a middle fossa approach was selected as the safest and most suitable option for implant placement. A myringotomy with transtympanic tube placement on the left was also performed.

**Figure 3:**
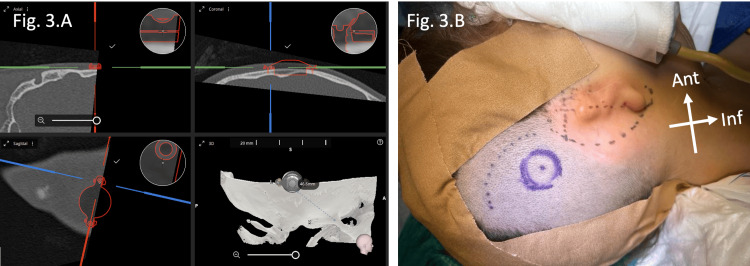
Surgical site planning (A) and markings (B) of Case 1. Modeling techniques with Otoplan® (CAScination AG, Bern, Switzerland) allowed for implantation using external landmarks, obviating the need for intraoperative image guidance.

Surgical planning and markings are illustrated in Figure 3, showing an anterior area designated for future pinna reconstruction, and a posterosuperior incision site for device implantation (Figure 3B).

Case 2

Patient Information

A 10-year-old female was referred to our hospital for hearing rehabilitation due to congenital unilateral HL on the left side. The patient reported difficulties in sound localization and hearing in noisy environments, which impacted daily communication.

Clinical Findings

On physical examination, she presented with complete atresia of the left external auditory canal, while the pinna was anatomically normal. Otoscopic examination of the right ear was unremarkable. No other craniofacial or systemic anomalies were identified.

Diagnostic Assessment

Audiometric evaluation revealed moderate conductive HL on the left side and normal hearing on the right. The PTA was 70 dB HL in the left ear and 13.75 dB HL in the right ear. Speech recognition thresholds were 75 dB HL on the left and 15 dB HL on the right, with 100% speech discrimination bilaterally.

**Figure 4:**
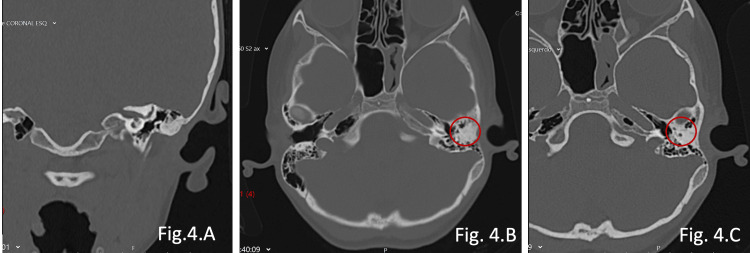
Temporal bone CT scan - coronal (A) and axial (B and C) views of Patient 2. A complete bony atresia of the external auditory canal (red circle), with a malformed middle ear on the left side, is seen. A very thin temporal bone is evident. The right ear anatomy is normal.

Temporal bone computed tomography demonstrated complete bony atresia of the left external auditory canal and a malformed middle ear (Figures 4A-4C). Additionally, markedly reduced temporal bone thickness in the middle fossa region was observed, limiting the feasibility of conventional mastoid implantation (Figure 4A).

Surgical Proposal

Given the insufficient bone thickness for standard implantation and the unfavorable local anatomy, a middle fossa approach was selected as the most appropriate and safe option for Bonebridge placement.

**Figure 5:**
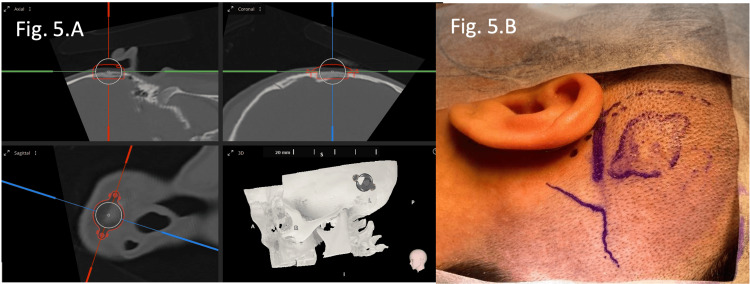
Surgical site planning and markings of Patient 2. (A) Modeling techniques with Otoplan® (CAScination AG, Bern, Switzerland) allowed implantation using external landmarks, obviating the need for intraoperative image guidance. (B) An anterosuperior incision was chosen for device implantation, as no skin preservation or pinna reconstruction was required.

Surgical site planning and markings are shown in Figure 5.

Surgical Technique

The decision to proceed with surgery was made following thorough discussion and informed consent from the patients' parents. The surgical technique was identical in both patients.

A semicircular incision was made around the planned implant site, superiorly in Case 1 (Figure 3B) and anterosuperiorly in Case 2 (Figure 5B). A double-layered flap consisting of skin and muscle was elevated to expose the temporal bone.

**Figure 6:**
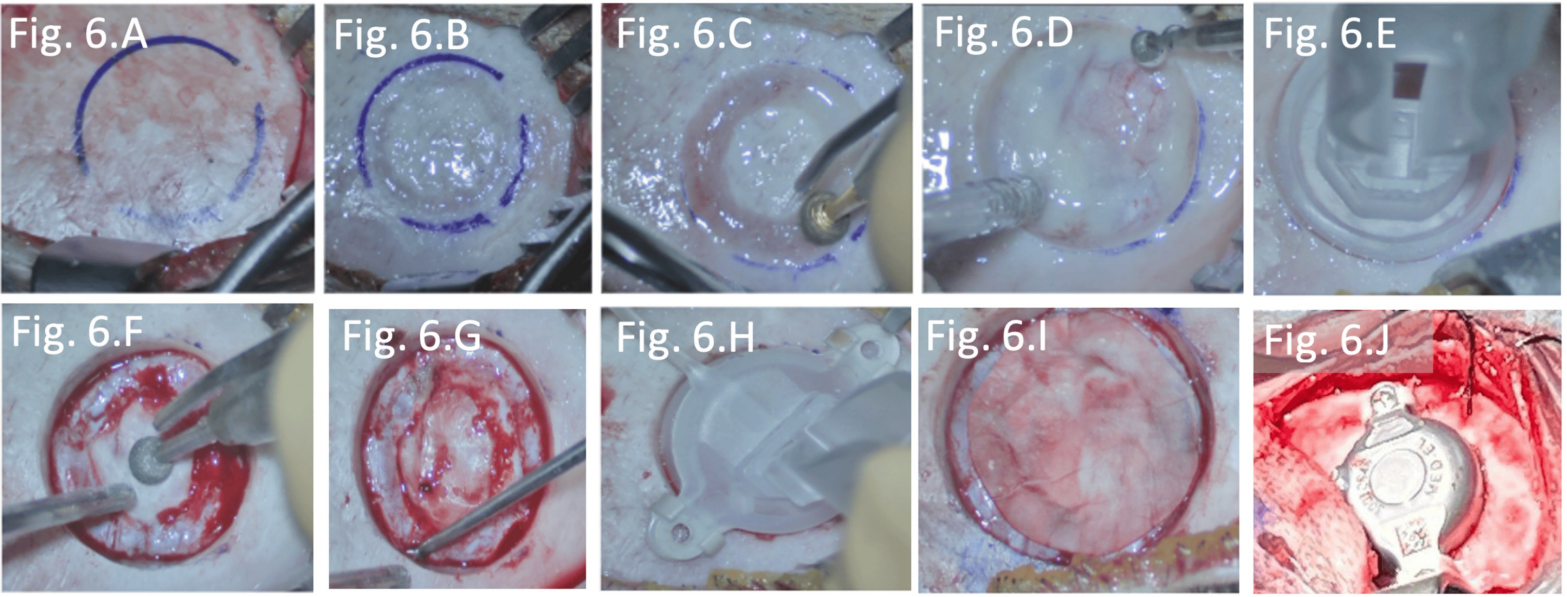
(A-I) Sequential drilling of the site for FMT placement and positioning of the dura substitute. (J) Final placement of the BCI 602 implant within the middle fossa cavity. The device is fully seated within the craniotomy, with surrounding bone edges smoothed and hemostasis achieved. It is elevated using 1-mm lifts and secured with titanium screws at an angled orientation for optimal adjustment to the thin temporal bone. Soft tissue coverage, including muscle and pericranium, is prepared to ensure optimal protection of both the implant and the underlying dura. FMT: floating mass transducer; BCI: bone conduction implant

The implant bed for the FMT was drilled using otologic burrs, exposing the dura of the middle cranial fossa (Figure 6). To prevent direct contact between the implant and the dura, an absorbable dural substitute was interposed (Figure 6I). In both cases, 1-mm lifts were used to reduce the required drilling depth and avoid dural compression. The BCI 602 implant was then positioned and secured with titanium screws placed at an angle to optimize adaptation to the thin temporal bone (Figure 6J). Finally, the muscle flap was repositioned to cover the implant, and the skin was closed using non-absorbable sutures. A compressive head bandage was applied on the first postoperative day.

No intraoperative complications were observed, including cerebrospinal fluid leakage or bleeding. Both patients remained stable during the postoperative period and were discharged home one day after surgery. At the one-week follow-up, surgical wounds were well healed in both cases.

Implant activation was performed one month after surgery using the SAMBA 2 audio processor (MED-EL, Innsbruck, Austria). Both patients achieved satisfactory auditory improvement. At one-year follow-up, the aided PTA improved to 32.5 dB HL in Patient 1, corresponding to a functional gain of 30 dB HL, and to 30 dB HL in Patient 2, corresponding to a functional gain of 40 dB HL.

Speech recognition thresholds improved to 40 dB HL in Patient 1 and 30 dB HL in Patient 2, with both patients achieving 100% speech discrimination at 60 dB and 55 dB, respectively. No device-related complications or adverse events were observed during the follow-up period.

A summary of clinical characteristics and outcomes is provided in Table 1.

**Table 1:** Summary of clinical characteristics, imaging findings, and outcomes. HL: hearing loss; EAC: external auditory canal; PTA: pure tone audiometry; SRT: speech reception threshold; BCI: bone conduction implant

Variable	Case 1	Case 2
Age/Sex	7-year-old female	10-year-old female
Condition	Goldenhar syndrome with microtia	Isolated unilateral atresia
Side affected	Right (severe HL), Left (mild conductive HL)	Left
Main complaint	Bilateral hearing loss, communication difficulties	Difficulty in sound localization and hearing in noise
Clinical findings	Right hemifacial microsomia and microtia, malocclusion, left effusive otitis media	Complete EAC atresia, normal pinna
PTA (unaided)	62.5 dB HL (left), 20.5 dB HL (right)	70 dB HL (left), 13.75 dB HL (right)
SRT (unaided)	65 dB HL (left), 20 dB HL (right)	75 dB HL (left), 15 dB HL (right)
CT findings	Thin temporal bone, emissary vein, atresia	Thin temporal bone, bony atresia
Reason for the middle fossa approach	Thin bone, vascular anomaly, need to preserve reconstruction site	Thin bone, unsuitable mastoid anatomy
Surgical approach	Middle fossa	Middle fossa
Implant	BCI 602	BCI 602
Follow-up	12 months	12 months
PTA (aided)	32.5 dB HL	30 dB HL
Functional gain	30 dB HL	40 dB HL
SRT (aided)	40 dB HL	30 dB HL
Speech discrimination	100%	100%
Complications	None	None

## Discussion

We report two pediatric cases of congenital ear malformations in which hearing rehabilitation was successfully achieved by placing the Bonebridge implant over the middle cranial fossa dura. These cases highlight the feasibility of this approach in anatomically challenging situations where conventional implantation sites are not suitable.

Although this surgical approach is considered off-label and remains relatively uncommon, its increasing use in recent years has led to a growing number of reports in the literature [6,8-11].

Zanetti and Di Berardino reported the use of Bonebridge in two patients with microtia and congenital stapes ankylosis [5]. Both patients experienced significant hearing improvement over a three-year follow-up, with excellent speech recognition scores and user satisfaction. In the same year, Der et al. presented their results in 24 pediatric patients with anatomical malformations who underwent Bonebridge implantation using the middle fossa technique [6].

Later, You et al. reported 40 cases of Bonebridge implantation using the middle fossa approach. No surgical complications were noted, and outcomes included favorable cosmetic results and a mean functional hearing gain of 39.6 dB (±14.7 SD) over a 29-month follow-up [10].

In 2021, Zernotti et al. introduced the Inverted Middle Fossa Approach (IMFA) as a novel surgical technique for Bonebridge implantation in patients with complex temporal bone anatomy, including aural atresia or syndromic malformations. Seven patients, aged 6 to 25 years, underwent this approach without intraoperative complications and with minimal postoperative issues [9]. Audiological outcomes were highly positive, with significant improvements in air conduction thresholds and speech discrimination. The IMFA also allows placement of the external processor closer to the ear canal, potentially enhancing sound localization and minimizing the shadow effect.

Currently, three t-BCI systems are commercially available: OSIA (Cochlear, Sydney, Australia), SENTIO (Oticon Medical AB, Gothenburg, Sweden), and Bonebridge. In our cases, the choice of device and technique was guided by specific anatomical factors, including limited cranial bone thickness, challenging mastoid anatomy (with one patient presenting a prominent emissary vein), and the need to preserve the surgical site for future auricular reconstruction. Therefore, a low-profile and flexible implant, suitable for placement at a distance from the auricle, was essential. The Bonebridge was therefore considered the most suitable option under these anatomical constraints.

The Bonebridge is a fully implanted transducer that transmits sound through mechanical vibrations via cortical fixation. In contrast to other osseointegrated systems, fixation does not rely on osseointegration, which may contribute to a shorter recovery period.

The BCI-602 implant has a reduced transducer thickness compared to earlier models, allowing implantation with less drilling depth and reducing the risk of compression of adjacent anatomical structures. This is particularly relevant in patients with limited temporal bone thickness [12,13]. In cases of reduced bone thickness, the dura may be exposed, and the use of lifts can help decrease the required bone bed depth, while avoiding dural compression [13,14].

Bae et al. described their experience with BCI-601 implantation in Korean children under the age of five [12]. While no intraoperative complications were observed, one patient developed increased intracranial pressure, which ultimately required explantation. The authors concluded that Bonebridge implantation may be safe and beneficial in this age group when performed with particular care to avoid sigmoid sinus compression.

The mastoid approach is the preferred route for Bonebridge placement in most patients when CT imaging reveals normal anatomical conditions [15-17]. However, in cases involving anatomical variants such as a low-lying dura, anteriorly positioned sigmoid sinus, post-surgical cavities, or, as in our case, a large emissary vein, this route may be contraindicated. In such scenarios, the retrosigmoid or middle fossa approaches are considered more viable alternatives [15]. The middle fossa approach has gained popularity very recently due to several notable advantages: it allows for a smaller incision within the hairline, offers better cosmetic outcomes, minimizes disruption of mastoid air cells and neck musculature, and significantly reduces surgical time [9,10,15]. Additionally, the middle fossa route avoids common risks associated with retrosigmoid placement, such as injury to the dura mater or sigmoid sinus [10]. In our patients, the presence of very thin temporal bones made the middle fossa approach particularly suitable.

Despite its advantages, the middle fossa approach for Bonebridge implantation entails specific anatomical and technical constraints. Adequate temporal bone volume is required to accommodate the implant without compromising surrounding structures. Additionally, there is a potential risk of dural compression, and the technique demands expertise in middle fossa anatomy and dural manipulation. A detailed anatomical evaluation using preoperative CT is essential for surgical planning. Critical parameters include bone thickness, dural height, and proximity to venous sinuses, all of which influence the selection of the optimal implant site [15]. The BCI-602 system is supported by Otoplan® software (CAScination AG, Bern, Switzerland), which enables precise preoperative assessment and helps anticipate anatomical challenges. In cases of thin temporal bone, 1-mm lifts may be used to prevent dural compression while maintaining secure implant fixation. Although there is a theoretical concern regarding headaches, cerebrospinal fluid leak, seizures, bleeding, or extradural hematoma caused by contact between the BC-FMT and the dura, no increased incidence has been reported in the literature [6,9]. Finally, the implant site in relation to the pinna must also be carefully considered, especially in pediatric cases. The cortical bone behind the pinna is typically avoided to minimize the risk of acoustic feedback, discomfort, and potential conflict with future auricle reconstruction. In cases involving congenital pinna malformations, implant placement should be meticulously planned to preserve tissue for future reconstructive procedures, emphasizing the need for individualized surgical planning. In the series of Der et al., no major complications were observed among the 24 children who underwent Bonebridge implantation [6]. Minor complications were documented in five cases, three of which involved patients with syndromic conditions. The most frequent issue following audio processor placement was localized skin erythema at the processor site, occurring in four patients, including one with Treacher Collins syndrome and another with Pierre Robin sequence. This was effectively resolved in all cases by reducing the magnet strength, with complete resolution within two weeks. One patient with Treacher Collins syndrome developed a postoperative scalp hematoma requiring needle aspiration on postoperative day 2. None of our patients had any of the complications described above.

The functional hearing improvements observed in our patients align with outcomes previously reported in the literature [6,8,14]. Bravo-Torres et al. reported a cohort of 15 children with congenital aural atresia who underwent Bonebridge implantation via the middle fossa approach [16]. A mean functional gain of 41.3 dB HL was observed postoperatively. Speech recognition scores improved from a preoperative mean of 29.4% to 96.4% at one month post-activation. Der et al. reported their audiological results on 24 children with atresia using the same technique. The functional gain of 35.5 dB HL in air conduction thresholds was reported, and the mean speech recognition score improved from 29.4% to 96.4% after a one-month follow-up [6]. Audiological outcomes reported in the literature for the middle fossa approach are comparable to those achieved with conventional placement [13,17]. Recent studies on bone conduction in humans have demonstrated that the cochlea can be stimulated via a fluid-mediated pathway [18]. Auditory brainstem responses have been elicited in neonates following bone conduction stimulation over the fontanelle, and audiometric responses have been observed in neurosurgical patients when stimulation was applied over a craniotomy. These findings support the feasibility of sound transmission through the middle fossa dura via both bone and soft tissue conduction.

This study has several limitations. First, it includes a small number of cases, which limits the generalizability of the findings. Second, follow-up duration is relatively short, and long-term outcomes remain to be established. Additionally, more comprehensive functional assessments, including speech perception in noise and patient-reported outcomes, were not systematically collected.

Further studies with larger cohorts and longer follow-up are needed to confirm the safety and effectiveness of this approach.

## Conclusions

Our findings suggest that middle fossa placement of the Bonebridge is a viable and promising alternative in selected pediatric patients with complex craniofacial anatomy. The functional outcomes were comparable to those seen in older patients, with no intraoperative or long-term complications observed.

Finally, it is also important to mention that the other transcutaneous implants, currently available on the market, with different features from the Bonebridge, may potentially be adapted to this approach in the middle fossa, if technical adaptations to the device and surgical technique are made. Further studies are warranted to validate the middle fossa dura as a reliable site for other transcutaneous bone conduction systems.
